# Persons with Dementia Living at Home or in Nursing Homes in Nine Swedish Urban or Rural Municipalities

**DOI:** 10.3390/healthcare7020080

**Published:** 2019-06-25

**Authors:** Connie Lethin, Ingalill Rahm Hallberg, Emme-Li Vingare, Lottie Giertz

**Affiliations:** 1 Department of Health Sciences, Faculty of Medicine, Lund University, 22100 Lund, Sweden; ingalill.rahm_hallberg@med.lu.se; 2 Department of Clinical Sciences, Clinical Memory Research Unit, Faculty of Medicine, Lund University, 21224 Malmö, Sweden; 3 Department of Social Work, Faculty of Social Sciences, Linnaeus University, 35195 Växjö, Sweden; emmeli.vingare@lnu.se (E.-L.V.); lottie.giertz@lnu.se (L.G.)

**Keywords:** dementia, dementia not otherwise specified, decision-making, healthcare, informal caregivers, informal caregiving, social services, quality of life

## Abstract

The methodology from the “RightTimePlaceCare” study of dementia care was tested locally in terms of relevance, acceptability and attrition. Comparing persons with dementia (PwDs) receiving home care (HC) with PwDs living in nursing homes (NHs), in urban versus rural areas, regarding their health conditions and informal caregiver burden was also done. Standardized measurements regarding sociodemographic, and physical and mental health was used. Questions related to legal guardianship were added. Interviews were conducted with PwDs and their caregivers in HC (n = 88) and in NHs (n = 58). Bivariate and multivariate logistic regression analysis was used. The attrition rate was higher in HC. In the bivariate regression model, for HC and NH, living at home was significantly associated with more severe neuropsychiatric symptoms (*p* ≤ 0.001) and being cared by a spouse (*p* = 0.008). In NH, the informal caregivers were significantly younger (*p* = 0.003) and living in rural areas (*p* = 0.007) and more often in paid work (*p* ≤ 0.001). In the multivariate regression model, informal caregivers were significantly younger (*p* = 0.007) when caring for a PwD in an NH and caregiver burden was significantly higher in HC and in urban areas (*p* = 0.043). Legal guardianship was very low. Professionals should acknowledge that PwDs in HC have more behavioural problems and caregivers in urban areas report higher caregiver burden.

## 1. Introduction

One major societal challenge in Sweden and worldwide is the rapidly growing older population implying an increasing prevalence of dementia and, consequently, a greater need for care [1,2,3]. Older people are at higher risk for various chronic diseases and dementia is among the most common age-related condition [4,5]. Persons with dementia have been shown to be more likely to have comorbidity complexes, such as Parkinson’s disease, stroke, diabetes, atherosclerosis, incontinence and pneumonia, which may stay undetected due to the dementia disease [6]. As the disease progresses, the person will be more dependent on informal and formal care to manage activities of daily living (ADLs) [7]. Neurocognitive symptoms occur throughout the dementia trajectory and the most common symptoms are agitation, mood disorders and psychosis [8]. Persons with dementia will develop one or more behavioural and psychological symptoms of dementia (BPSD) with an impact on quality of life (QoL) and caregiver burden. At some point also depressive symptoms occur during the course of dementia (close to 80% of all persons with dementia) and some of them may have a major depressive disorder (10–20%) [9]. The progressive course of the disease requires a well-elaborated chain of care to achieve optimal QoL for persons with dementia and also to support the informal caregiver.

Informal caregivers are often the main provider of care and service for persons with dementia living at home [10,11]. They may experience the caregiving as positive [12] but are also at increased risk for burden, stress, depression and other health complications [11,13]. A challenge for professionals in dementia care is to deliver timely, individualized quality care and support both in urban and in rural areas and to follow up the care and treatment for persons with dementia and their informal caregivers. Depending on how public responsibility is distributed, there may be difficulties in rural municipalities to provide sufficient care due to financial strain. Also, there is a need for methods to follow up the quality of care that can be feed-back to the public providers.

Because of an increase in cognitive impairment, having dementia disease means intrusion into a person’s life and the life of the people surrounding the person [14,15]. Due to the detrimental impact on cognitive ability from the disease, there is also a need to explore how legal aspects are handled within municipal care and services, including for instance, whether guardianship is established. Dementia diseases are progressive which implies that the need for healthcare and social services (care and services) changes throughout the course of dementia. It is well known that the opportunity to move into a nursing home is limited meaning that several persons with severe dementia is cared for at home. This may cause strain on the informal caregivers. The knowledge is scarce, both regarding the effort needed through the course of the disease and regarding the timing when care in ordinary housing or residential housing becomes appropriate. Knowledge is also limited regarding how to design support for the family that has responsibility for the person’s everyday care [16].

In the European (EU)-funded study RightTimePlaceCare (RTPC) the care and service systems, from diagnosis to end of life, in eight European countries were investigated between 2010 and 2013 to capture the national situation regarding dementia care, compared between the countries and develop best practice [17]. The RTPC study consisted of two parts: an interview study exploring the living conditions of persons with dementia and their informal caregivers living at home with home care or living in nursing homes and a mapping study exploring the care and service systems available [18]. The project titled “Living with dementia, care and service systems” (also known as the LwD-study) was inspired by the RTPC project and its main objective was to develop best practice strategies for dementia care locally. The mapping system was tested and further developed in the LwD study for adaption to a local Swedish context. The mapping system was tested and further developed in the LwD study for adaption to a local Swedish context and was found useful and reliable [19].

In this part of the LwD study, the circumstances and living conditions of persons with dementia receiving formal and informal care when living at home or in nursing homes as well as conditions for their informal caregivers were investigated. This was so since it is the main responsibility of the local providers. Furthermore, we studied factors influencing the person with dementias institutionalization at the time of admission to a nursing home and the availability and utilization of care and services throughout the dementia trajectory as well as professional providers’ educational level. Emphasis was on QoL and the quality of care of persons with dementia and informal caregiver burden and QoL of informal caregivers of persons with dementia in nursing homes and home care. The LwD-study generated primary data at a local level on the transition from professional home care to nursing homes for persons with dementia and their informal caregivers in nine municipalities, urban and rural. The expectations were that this study would make the professionals aware of the needs of the individual as well as their informal caregivers and of problems that need to be alleviated. The first aim of this study was, in collaboration with professionals, to adapt the methodology from a European study to local circumstances in terms of relevance, acceptability and attrition (the feasibility) of the methodology in nine Swedish municipalities. The second aim was to compare persons with dementia living at home to persons with dementia living in nursing homes regarding, sociodemographic status, physical and mental health, independence, behavioural symptoms and the question of guardianship. For their informal caregivers, sociodemographic circumstances, paid work and caregiver burden were investigated. The third and final aim was to compare persons with dementia living at home to persons with dementia living in nursing homes and to compare conditions for their informal caregivers in urban versus rural municipalities regarding the same outcomes as for the second aim.

## 2. Materials and Methods

### 2.1. The Swedish Context

In Sweden, the care of persons with dementia is a shared responsibility between the counties and the municipalities. The county councils are responsible for health care in accordance with the Health and Medical Services Act [20]. Medical healthcare such as screening, dementia diagnosis and medical treatment is available at primary healthcare centres or specialized clinics at hospitals and is mainly provided by the county councils.

In terms of the Social Services Act [21] municipalities have the responsibility for care of the citizens who are 65 years or older. The municipalities are responsible for general and specialized care and services in home care and nursing homes and also for respite care and day care. They are expected to enable older people to stay in their ordinary housing for as long as possible under safe circumstances supported with care and services whenever needed. Social services also have a special responsibility to support the informal caregivers. Both healthcare and social services in Sweden are publicly funded through taxes. This in turn means that the tax income in rural areas is dependent on people in the work force and if a large share is retired or not in working the municipality will have difficulties to provide the care and support needed for their older population. The distribution can be by public authorities although there has been an increase in marketization during the last decade. All assessment is based on voluntary participation with consent from the care and service recipient.

### 2.2. Design

This cross-sectional study was conducted in nine municipalities situated in two counties in the south of Sweden. The municipalities were invited to a meeting regarding this project. Written information describing the LwD project was sent to all executives of eldercare in one county. All eight municipalities within one county participated, all falling under the same healthcare organization. The ninth municipality was located in the closest neighbouring county and the meeting there was initiated by the local authorities. Written consent, with a mutual responsibility to contribute to the research, was obtained from the participating municipalities.

### 2.3. Adaption of the Methodology

The study was adapted to Swedish conditions to enable comparison between the municipalities, illuminate available resources and to give feedback to politicians and people in charge of the care and service system in the municipalities. A reference group was established with representatives from all participating municipalities consisting of professionals, such as social workers, registered dementia nurses and eldercare managers and care coordinators. The entire project was discussed, including; recruiting process; the relevance of the questions included in the interview and the acceptability of the procedure. In addition, questions about legal guardians were brought up, along with other questions of interest. To achieve consensus, the research team and the reference group had face-to-face meetings four to five times a year complemented by e-mail messages.

Since aspects related to legal guardianship is the responsibility of the municipality questions about how and by whom this was handled. Persons with extensively reduced decision-making ability may need assistance and support from a substitute decision-maker and legal representative in their contact with the care and service systems as well as of handling their economic and legal affairs. Legal guardianship is a responsibility of the municipality following a district court judgment. It is a formal response to the person’s impaired decision-making ability through the appointment of a substitute decision-maker. Guardianship can include handling of the client’s finances and monitoring of his or her legal rights. According to the Parental Code [22] the municipality has the responsibility to recruit and control legal guardians and assist the district court in the investigation before judgment is made.

### 2.4. Procedure

Baseline and follow-up interviews were conducted with persons with dementia and their informal caregivers from March 2014 to July 2016. A follow-up interview was performed 80–100 days after the baseline interview, if the person or caregiver agreed to this. Nine months after the baseline interview, the contact person in each participating municipality made a report on the living situation of the person with dementia regarding survival and present housing. All instruments used were selected based on their psychometric properties and appropriateness for the aim and population. Questionnaires were translated into Swedish according to a standardized procedure from the RTPC project [17]. Professionals (registered dementia nurses, registered nurses, social workers and eldercare managers) were asked to recruit possible participants after being informed about inclusion and exclusion criteria and instructed how to inform persons with dementia and their informal caregivers about the LwD project. To standardize the data collection and assure data quality, a manual was developed consisting of two parts: preparation for the interviews with information about the selection of institutions and participants and instructions for interviewers; and the interview content, explaining the measurement assessments used during the interviews and current ethical codes. Furthermore, special training was provided to the professionals by the researches. The trained interviewers collected all data performing face-to-face interviews. In 2015, three students in social work or nursing and one social worker were trained as interviewers and conducted altogether 17 interviews.

### 2.5. Recruitment of Participants

Inclusion criteria for persons with dementia were being 65 years of age or older, having a primary dementia diagnosis, scoring 24 or lower on the Standardized Mini-Mental State Examination (S-MMSE) [23] and having an informal caregiver visit at least twice a month. The number of informal caregivers was limited to one main caregiver, defined as the person who was most involved in the care of the person with dementia (partners, adult children, other relatives or others such as friends or neighbours). Persons with dementia (and their informal caregivers) who were between the margins of care were eligible for the study; that is, persons who were at the point when home care may become insufficient or inadequate and who were facing admission to a nursing home within 6 months. This process may be initiated when the informal caregiver can no longer handle the situation or the person with dementias care dependency increases and the needs cannot be sufficiently met by community services or informal care. The judgment of risk was made by a professional caregiver for example, a registered dementia nurse or a social worker.

Also eligible for the study, were persons with dementia who had recently been judged eligible for nursing home care. In the research design, the plan was to include 85 persons with dementia and informal caregiver dyads in home care and 60 dyads in nursing homes. The interviews in the nursing homes were conducted 1–3 months after the person with dementia had moved there. Persons with dementia eligible for nursing home care for a limited period to provide respite care at home were excluded. At the first contact, professionals in the municipalities proposed 175 persons with dementia–caregiver dyads from both urban (≥20,000 inhabitants) and rural areas (<20,000 inhabitants), to participate in this study and were given verbal informed consent. At the second contact by researchers, 146 (83%) persons with dementia and their informal caregivers chose to participate in the study and gave written informed consent (see Supplementary file of descriptive data of the inhabitants in the participating municipalities and the study’s attrition rate). At baseline, 88 interviews were conducted with persons with dementia and their informal caregivers in home care and 58 interviews in nursing homes. Altogether 274 interviews were conducted, at baseline and at a follow up after 3 months.

### 2.6. Measurements

Before each interview, the cognitive function of the person with dementia was tested to meet the inclusion criteria (Table 1). Sociodemographic information on persons with dementia included age, gender, dementia diagnosis, marital status and living situation for the persons with dementia (i.e., living at home versus in a nursing home). Other measurements for persons with dementia were rated by proxy (informal caregivers in home care or formal caregivers in nursing homes) regarding QoL and physical and psychological health. Measurements for informal caregivers included sociodemographic information regarding age, gender, marital status, having paid work and relationship to the person with dementia (not for nursing homes). Furthermore, the interviews contained questions regarding caregiver burden, QoL and the use of legal guardianship. See Verbeek et al. [17] for a more detailed description of measurements.

### 2.7. Analysis

Bivariate logistic regression analysis was performed for associated factors for persons with dementia living at home or in nursing homes and in urban versus rural areas. As the intention was to conduct 13 separate analyses for persons with dementia, we adjusted using Bonferroni correction, therefore for this analysis *p* ≤ 0.003 was regarded as significant. For informal caregivers, the intention was to conduct 6 separate analysis and when adjusted using Bonferroni correction, *p* ≤ 0.008 was regarded as significant. Backward stepwise multivariate regression analysis was performed for urban and rural areas. For the multivariate regression analysis, a *p*-value of *p* ≤ 0.05 was regarded as significant. For all analyses, SPSS version 25.0 was used (IBM Corp., Armonk, NY, USA). For the analysis, the variables urban (municipalities 2, 5, 6, 7 and 9) and rural areas (municipalities 1, 3, 4 and 8) were dichotomized into 1 = urban area and 0 = rural area.

### 2.8. Ethical Approval

For ethic approval and consent to participate, the procedures followed the ethical considerations in human experimentation [24] and the Declaration of Helsinki [25]. The LwD project was granted ethical approval by the Regional Ethical Review Board, Lund, Sweden (Dnr 2010/538; 2014/168). Persons with dementia and their informal caregivers participated voluntarily and in a first stage they gave informed verbal consent to professionals, allowing the researchers to contact them. Prior to the interview, they signed a written informed consent form and if possible, this was signed by the person with dementia themselves, otherwise by their informal caregiver as a proxy. Persons with dementia who were unable to sign informed consent were asked to assent. “Assent” was defined as willingness to participate even without full understanding of the complexity of the study. During the interviews, a sense of comfort for the participants, with active monitoring of their willingness to participate and of any signs of distress, was provided by the interviewers. An external audit of data plausibility was performed to ensure the quality of data collection and data management. The external auditor holds a Ph.D. in nursing science and was not involved in the study. The data check covered 10% of randomly selected protocols.

## 3. Results

### 3.1. Adaption of a European Study in Nine Swedish Municipalities

To adapt and facilitate the data collection the reference group met several times. During January to March 2014, the research team adapted the interview protocol into local conditions, which implied minor changes. In the interview study, one question was removed regarding restraints methods, since this method is not eligible in Sweden. For the instrument regarding resource utilization in dementia care (RUD), two questions about country and ethnicity were removed regarded as not relevant. Three questions about informal caregivers’ healthcare contacts were removed as they were not regarded as relevant for the study and had previously not been answered in the European study. It was agreed to include questions regarding guardianship and the decision-making of persons with dementia to investigate the use of formal and informal guardianship. The questions added were open semi-structured questions and questions with fixed answer options and were answered by the informal caregiver. The questions covered decision making in daily life and the delivered care and service from the municipality. In addition to these changes, some editorial changes were made. The process of inclusion and recruitment was regarded acceptable and agreed on. 

In the two counties, the urban area was a one midsized city and two small towns as defined by Statistics Sweden. Four municipalities were rural areas with one or more central villages. In the nine municipalities, the population ranged between 8256 and 86,970. Despite the difference, the municipalities were fairly similar regarding the distribution of people aged 65–84 years. In rural areas, inhabitants 65 years or older made up between 20 and 26% of the population and inhabitants 85 years or older accounted for 3–4%. Most inhabitants in the smallest municipalities were living in small villages not located in the countryside but still considered rural areas. In urban areas, inhabitants 65 years or older constituted between 18% and 28% of the population and 3–5% were 85 years or older. The attrition to the study was higher in home care (83–100%) compared to nursing homes (55–60%).

### 3.2. Associated Factors for Persons with Dementia Living at Home or in a Nursing Homes

There were more men (59%) with dementia living at home compared to living in a nursing home (not significant, NS) (Table 2). In nursing homes, on the other hand, there were more women (59%) than men (NS). An adult child significantly more often cared for the persons with dementia living at home. The most common diagnosis was unknown dementia type; 53% of the persons with dementia living in nursing homes and 35% of the persons with dementia in home care did not have a specified dementia diagnosis (NS). Persons with dementia living at home had significantly more severe neuropsychiatric symptoms and informal caregivers had significantly higher experienced burden, compared to those living in nursing homes. There were marginal differences in comorbidity, QoL and independence in activities in daily life between persons with dementia in the two living conditions. The informal caregivers participating in the study were more frequently spouses (63%) and significantly more often wives than adult children (29%), caring for persons with dementia living at home. In nursing homes, the most common informal caregiver was the person with dementias adult child (62%, NS) and significantly more often in paid work (55%). Legal guardianship was not utilized to any extent; only eight out of 146 persons with dementia had a legal guardian or another person acting as guardian’ and out of those eight, most were living at home. Most informal caregivers had a power of attorney to manage financial affairs on behalf of the person with dementia, 91% in home care and 94% in nursing homes (NS) (Table 2).

### 3.3. Associated Factors for Persons with Dementia Living in Urban versus Rural Municipalities

In the bivariate regression analysis, informal caregivers were significantly younger when caring for a person with dementia living at home in rural areas, compared to the informal caregivers in urban areas (Table 3). There was no association between urban and rural areas regarding variables such as gender, cognition, comorbidity, QoL and physical or psychological health.

### 3.4. Associated Factors in the Multivariate Regression Analysis

In the multivariate regression analysis for persons with dementia, living at home or in a nursing home there was no association with any of the variables (not presented in any Table). Urban areas were associated with higher caregiver burden when caring for a person with dementia living at home, compared to living in rural areas (Table 4).

## 4. Discussion

The RTPC interview study needed some minor adjustments to be adapted to local conditions of dementia care in nine municipalities of different size. Thus, it seems plausible that the methodology would be adaptable to other local conditions in other parts of Sweden as well as in other countries and thus measuring the person with dementias situation and care at the level closest to the responsible organization. The strengths of the study were that it was conducted in collaboration with local authorities and staff at various levels. The involvement of participants in the municipalities was an essential part of planning for this study and the interest and involvement differed between municipalities.

The main objective of the LwD project was to develop knowledge that could be used to improve healthcare and social services for citizens with dementia and develop best practice strategies’ for individualized care. The ongoing discussions in the reference group and the feedback to managers in dementia care in the nine municipalities were an important step starting this work in dementia care. In some of the municipalities, the user organizations took advantage of the project to highlight the hard and difficult work that informal caregivers do in every-day life, by inviting politicians and informal caregivers for an information and discussion session in which the work of the in the LwD project was the point of departure. Recruitment of participants in our study, involved inviting 175 persons with dementia and their informal caregivers to participate in the LwD study and 83% choose to participate at baseline (n = 146).

Most noteworthy was perhaps the fact that legal guardianship was not implemented to any extent, it was in most cases family members who informally took care of legal and financial issues and this raises questions about legal certainty and risk of economic abuse. Dementia is characterized by a progressive deterioration in cognitive abilities and incapacity for decision-making and independent living [15]. Financial abuse of older people is a serious and common problem [26] and persons with dementia particularly vulnerable to it [27]. This increases the need for support in decision making and handling of personal affairs as persons with dementia may need increasing support in their daily living and with decision making and executive decisions. This raises the important question of autonomy but also the question of the protection of legal rights. In Sweden and other countries, there are various formal and informal legal support networks. In many countries, legal personal representatives such as trustees, limited guardians or legal administrators are appointed by court to legally represent and act for a person with limited ability [4] and protect the rights of the individual. There is also power of attorney, which primarily regulates the individual’s financial situation [28]. In health care, there are possibilities for surrogate decision making. The questions in this study were developed to capture an understanding of the supported and substituted decision-making experience. All types of decision making affect daily life and contacts with the social welfare system and other authorities and are therefore a growing issue in all Western societies. Vulnerability is reinforced with an increasing emphasis on the client’s rights and the idea of free choice. In reality, the rights and freedoms of elderly people with progressing dementia are fragile [29]. Scholars from different fields of research have found that decision making can be an oppressive aspect of caregiving for persons with dementia and this question is debated in all Western countries [30,31]. More knowledge is needed about decision making and legal protection in dementia care. This study may be the beginning of a broader discussion of issues related to informal and formal decision making regarding persons with dementia.

Gender may prove to be related to access to care and service systems. Earlier research in Sweden paid attention to gender inequality in utilization of and access to, social care support [32]. The present study confirms that there are more women than men who care for their partner in ordinary housing, as shown by previous Swedish research [32]. One reason for this may be that women usually marry men older than themselves and that men die younger than women. Participants in this study (n = 88) were largely men, with the wife being the informal caregiver. Persons with dementia who had recently moved to nursing homes were mostly women (59%). This raises questions about how support efforts are distributed and for what reasons. It also raises the question whether today’s chain of care provides gender-biased support. Instrumental ADLs in ordinary housing are provided for “the traditionally female jobs,” like cooking and cleaning, while taking care of the car and other “male jobs” are not included. This way, female caregivers become more vulnerable because there is no support in areas where they might need it. Also, husbands as caregivers may be more likely than wives to seek help from formal care and receive more support but this is subject for further studies.

Informal caregivers in urban areas, compared to caregivers in rural areas, seem to experience higher burden when caring for a person with dementia living at home. This is contradictory, as care and services are provided to a higher extent in urban areas compared to rural areas, as reported by Moholt et al. [27]. Their results showed that the use of home-based services among persons with dementia was significantly higher for persons living in urban areas and it could be assumed that this would ease, rather than exacerbate, the caregiver burden. In their study, use of care and service support, which may have explained the differences, was not investigated. When we previously explored the availability and utilization of dementia care throughout the dementia trajectory in the LwD study, we found no difference between urban and rural areas [15]. There is no indication in our study that there any difference between urban and rural municipalities providing formal care with impact on the measures used in this study. However, to our knowledge, no studies have investigated differences in caregiver burden between urban and rural areas in Western countries and this need to be further explored.

Persons with dementia living at home seem to report more severe neuropsychiatric symptoms. Our study showed that neuropsychiatric symptoms were significantly more common in persons with dementia living at home. On the one hand, this may be related to not having a specific dementia diagnosis and appropriate pharmacological treatment. Acetylcholinesterase inhibitors and memantine as pharmacological treatment are specific for neurodegenerative diseases such as Alzheimer’s disease and Lewy body dementia and are believed to ameliorate neuropsychiatric symptoms in dementia even though the evidence is contradictory [31]. On the other hand, it may also be related to health literacy and willingness to seek care as well as that the caregiver burden may be too difficult to handle and thus creating a hostile climate in the home. The person and the informal caregiver need to be able to recognize the need for care and services. This ability is dependent on components such as health literacy and own health and sickness-related beliefs [32]. Appropriate pharmacological treatment and psychosocial interventions are essential for both the person with dementia and the informal caregiver since neuropsychiatric symptoms are known to decrease the QoL of both the person with dementia [33] and the caregiver, as well as increase the caregiver burden [15].

Dementia not otherwise specified (DNOS) may be related to the organization of health care in each municipality and to cooperation between the county councils and municipalities. The results in this study showed that, in the nine participating municipalities, there were many persons with DNOS (35%) living at home and the number was even higher if living in a nursing home (53%) but without significance. This confirms previous studies that also show a higher level of DNOS in nursing homes [28] compared to home care [29,30]. This may suggest that dementia investigations and diagnoses are not fully developed in the participating municipalities and the health care system. Having an accurate diagnosis affects the person’s possibility to receive adequate treatment and support [34]. The consequences for persons having a specific dementia diagnosis may be that they will not be able to plan for the future and they may suffer from missed opportunities for pharmacological treatment and psychosocial support for dementia and support, such as dementia day care centres, which require a diagnosis. This issue is of interest for further research.

The strength of this study is the collaboration with the reference group of representatives from all participating municipalities, consisting of professionals such as social workers, registered dementia nurses, eldercare managers and care coordinators. Furthermore, we conducted interviews with a group of persons with dementia at risk of moving to a residential home and their informal caregivers, which made it possible to find associated factors for the transition from home care to nursing homes. The project used the same guidelines and manuals for all municipalities and researchers, which strengthens the internal validity. The study investigated care in both urban and rural areas within the same data collection, which provides external validity. Limitations in this study are the fact that this was a group of persons with dementia who were at risk of moving to a residential home and that the results are not representative of persons with dementia in general. On the other hand, this was a population-level study including nine participating municipalities.

## 5. Conclusions

The main contribution of this study is that it shows the possibility to adapt a European methodology to a local municipality level. The interview study needed some minor adjustments for adaptation to a local level and it seems plausible that it would be adaptable for use in other countries and other health care systems. For professionals to provide adequate treatment, they should acknowledge that persons with dementia, living at home seem to have more behavioural problems and depressive symptoms compared to those living in nursing homes; also, informal caregivers in urban areas may experience higher caregiver burden. Most noteworthy is the fact that legal guardianship was not utilized to any extent. In most cases, family members informally took care of legal and financial matters, which raises questions about legal certainty and the risk of economic abuse.

## Figures and Tables

**Table 1:** Measurements included and related to the situation of the person with dementia.

Variable	Assessment	Measure	No. of Items
ADLs	Proxy	Katz Index of Independence in ADLs (Katz-ADL)	6
Behaviour	Proxy *	Neuropsychiatric inventory questionnaire (NPI-Q)	12
Caregiver burden	Informal caregiver	Zarit Burden Interview (ZBI)	22
Cognition	Person with dementia	Standardized Mini-Mental State Examination (S-MMSE)	20
Comorbidity	Proxy	Charlson Comorbidity Index (CCI)	n/a
Depression	Proxy	Cornell Scale for Depression in Dementia (CSDD)	19
Guardianship	Proxy	Datasheet	21
Quality of life	Person with dementia and proxy	Quality of life in Alzheimer’s disease (QoL-AD)	13
Socio-demographics	Proxy	Datasheet	n/a

ADL = activities of daily living; n/a = not applicable. * Answered by informal caregivers at home and formal caregivers in nursing homes.

**Table 2:** Bivariate regression analysis of persons with dementia living in home care versus in a nursing home and their informal caregivers.

Person with Dementia and Caregivers	HC (n = 88)	NH (n = 58)	OR	95% CI	*p* *
Person with dementia (PwD)					
Age in years, median, (Q1, Q3)	80 (76, 87)	84 (79, 88)	0.946	0.898–0.997	0.040
Female gender, n (%)	36 (41)	34 (59)	0.489	0.249–0.958	0.037
Dementia symptoms, years, median, (Q1, Q3)	5 (3, 7)	5 (3, 7)	1.020	0.920–1.131	0.704
Cognitive function, (S-MMSE), 0–30	16 (10, 20)	11 (7, 18)	1.016	0.967–1.068	0.535
Dementia diagnosis, n (%)					
Alzheimer´s disease (reference value)	20 (23)	13 (22)			0.250
Alzheimer´s disease/vascular dementia	6 (7)	2 (3)	1.950	0.340–11.177	0.453
Vascular dementia	22 (25)	6 (10)	2.383	0.761–7.461	0.136
Frontotemporal dementia	2 (2)	2 (3)	0.650	0.081–5.206	0.685
Lewy body dementia	3 (3)	4 (7)	0.487	0.093–2.543	0.487
Unknown dementia type	31 (35)	31 (53)	0.650	0.276–1.532	0.650
Other	4 (5)	0 (0)	105005864	0.000	0.999
Comorbidity (CCI), 0–37, median, (Q1, Q3)	2 (1, 3)	1 (1, 2)	0.969	0.873–1.076	0.560
Quality of life (QoL-AD), 13–52	33 (29, 37)	35 (31, 38)	0.951	0.867–1.044	0.293
Activities of daily living (Katz ADL), 0–6	4 (2, 5)	4 (2, 5)	–	–	0.411
Neuropsychiatric symptoms (NPI-Q)					
Severity, 0–36	10 (7, 15)	7 (3, 10)	1.119	1.046–1.197	**<0.001**
Depression (CSDD), 0–38	5 (0, 8)	0 (0, 5)	1.113	1.019–1.216	0.018
Guardianship, n (%)					0.850
Legal guardianship	4 (5)	1 (2)	111063895	0.000	1.000
Guardianship, other person	1 (1)	2 (3)	2.750	0.299–25.270	0.371
Power of attorney for finaces	80 (91)	55 (94)	0.000	0.000	0.999
Informal caregivers of the PwD					
Age in years, median, (Q1, Q3)	73 (65, 78)	65 (55, 73)	1.045	1.015–1.076	**0.003**
Female gender, n (%)	71 (81)	41 (70)	1.732	0.798–3.757	0.165
Paid work	18 (21)	32 (55)	0.209	0.100–0.434	**<0.001**
Cohabiting with the PwD	56 (64)	–	–	–	–
Relation to the PwD					**<0.001**
Husband (reference value)	16 (18)	5 (9)	0.938	0.287–3.064	0.915
Wife	39 (45)	13 (22)	0.217	0.070–0.669	**0.008**
Adult child, son/daughter	25 (29)	36 (62)	0.547	0.112–2.673	0.456
Friend	0 (0)	0 (0)	–	–	–
Other	7 (8)	4 (7)	–	–	–
Caregiver burden (ZBI), 0–88, median, (Q1, Q3)	39 (28, 49)	24 (18, 37)	1.057	1.029–1.086	**<0.001**

CCI = Charlson Comorbidity Index; CI = confidence interval; CSDD = Cornell Scale for Depression in Dementia; Katz-ADL = Katz Index of Independence in Activities of Daily Living; n = number of participants; NPI-Q = Neuropsychiatric Inventory Questionnaire; OR = odds ratio; Q1 = first quartile; Q3 = third quartile; QoL-AD = Quality of life in Alzheimer’s disease; S-MMSE = Standardized Mini-Mental State Examination; ZBI = Zarit Burden Interview. Underlined values indicate positive results, for example, 0–30. * For persons with dementia, *p* < 0.003 was regarded as significant and for informal caregivers *p* < 0.008 was regarded as significant; significant *p*-values are marked in bold.

**Table 3:** Bivariate regression analysis and associated factors of persons with dementia and informal caregivers in urban versus rural areas.

Person with Dementia and Caregivers	Living at Home					Living in a Nursing Home				
	Urban, n = 55	Rural, n = 33	OR	95% CI	*p*	Urban, n = 33	Rural, n = 25	OR	95% CI	*p*
Person with Dementia										
Age in years, median, (Q1, Q3)	79 (75, 86)	81 (77, 8)	0.958	0.896–1.024	0.209	86 (80, 89)	83 (76, 87)	1.064	0.976–1.160	0.162
Female gender, n (%)	21 (38)	15 (46)	0.741	0.309–1.778	0.502	18 (55)	16 (64)	0.675	0.232–1.960	0.470
Dementia symptoms, years, median, (Q1, Q3)	4 (3, 7)	5 (4, 8)	0.978	0.861–1.111	0.737	6 (3, 9)	4 (2, 6)	1.127	0.939–1.353	0.200
Cognitive function (S-MMSE), 0–30	16 (10, 21)	16 (9, 20)	1.044	0.970–1.124	0.252	11 (6, 17)	13 (9, 21)	0.938	0.829–1.061	0.309
Dementia diagnosis, n (%)										
Alzheimer´s disease (reference value)	16 (28)	4 (12)			0.591	9 (27)	4 (16)			0.805
Alzheimer´s disease/vascular dementia	3 (6)	3 (9)	0.250	0.036–1.739	0.161	0 (0)	2 (8)	0.000	0.000	0.999
Vascular dementia	13 (24)	9 (27)	0.361	0.090–1.445	0.150	2 (6)	4 (16)	0.222	0.028–1.754	0.154
Frontotemporal dementia	2 (4)	0 (0)	4038687	0.000	0.999	1 (3)	1 (4)	0.444	0.022–9.032	0.598
Lewy body dementia	2 (4)	1 (3)	0.500	0.036–6.997	0.607	2 (6)	2 (4)	0.444	0.045–4.374	0.487
Unknown dementia type	16 (28)	15 (46)	0.267	0.072–0.981	0.047	19 (58)	12 (48)	0.704	0.177–2.802	0.618
Other	3 (6)	1 (3)	0.750	0.061–9.270	0.823	0 (0)	0 (0)	–	–	–
Comorbidity (CCI), 0–37, median (Q1, Q3)	1 (1, 2)	2 (1, 3)	0.931	0.726–1.194	0.574	1 (1, 2)	1 (1, 4)	1.015	0.902–1.142	0.800
Quality of life in (QoL-AD), 13–52	33 (29, 37)	33 (29, 36)	1.033	0.934–1.142	0.532	35 (31, 38)	34 (31, 38)	1.024	0.866–1.024	0.784
Activities of daily living (Katz ADL), 0–6	4 (2, 5)	4 (2, 5)	1.107	0.818–1.498	0.511	4 (2, 5)	4 (2, 5)	1.004	0.720–1.401	0.981
Neuropsychiatric symptoms (NPI-Q)										
Severity, 0–36	10 (6, 16)	10 (7, 14)	1.004	0.937–1.077	0.905	7 (4, 11)	6 (3, 9)	1.063	0.955–1.183	0.264
Depression (CSDD), 0–38	3 (0, 7)	5 (1, 10)	0.953	0.871–1.043	0.295	0 (0, 7)	1 (0, 3)	1.122	0.951–1.324	0.172
Guardianship, n (%)										
Legal guardianship	3 (6)	1 (3)	1.000	0.000	1.000	1 (3)	0 (0)	1.000	0.000	1.000
Guardianship, other person	1 (2)	1 (3)	1.846	0.184–18.518	0.602	3 (9)	25 (100)	1.320	–	0.295
Power of attorney for finaces	38 (79)	25 (78)	0.940	0.316–2.795	0.911	27 (96)	21 (88)	0.259	0.025–2.675	0.257
Informal caregivers of the PwD										
Age in years, median, (Q1, Q3)	72 (69, 78)	72 (69, 78)	1.021	0.981–1.062	0.309	68 (59, 77)	55 (50, 72)	1.072	1.019–1.127	**0.007**
Female gender, n (%)	43 (78)	28 (85)	1.563	0.496–4.919	0.445	25 (76)	16 (64)	1.758	0.562–5.499	0.332
Paid work	9 (16)	9 (27)	0.522	0.183–1.487	0.223	17 (52)	15 (60)	0.708	0.247–2.028	0.520
Cohabiting with the PwD	38 (70)	18 (55)	1.863	0.763–4.547	0.172	–	–	–	-	-
Relation to the PwD										
Husband (reference value)	10 (19)	6 (18)			0.164	3 (9)	2 (8)			0.280
Wife	28 (52)	11 (33)	1.527	0.447–5.221	0.500	10 (30)	3 (12)	2.222	0.245–20.174	0.478
Adult child, son/daughter	11 (20)	14 (42)	0.471	0.131–1.702	0.251	17 (52)	19 (76)	0.596	0.089–4.008	0.595
Friend	0 (0)	0 (0)	1.500	0.218–10.304	0.680	0 (0)	0 (0)	–	–	–
Other	5 (9)	2 (6)	–	–	–	3 (9)	1 (4)	2.000	0.218–10.304	0.638
Caregiver burden (ZBI), 0–88, median (Q1, Q3)	44 (31, 51)	34 (34, 46)	1.033	1.001–1.066	0.043	22 (15, 37)	27 (22, 35)	0.389	0.943–1.023	0.389

CCI = Charlson Comorbidity Index; CI = confidence interval; CSDD = Cornell Scale for Depression in Dementia; Katz-ADL = Katz Index of Independence in Activities of Daily Living; n = number of participants; NPI-Q = Neuropsychiatric Inventory Questionnaire; Q1 = first quartile; Q3 = third quartile; QoL-AD = Quality of life in AD; S-MMSE = Standardized Mini-Mental State. Underlining of values indicates a positive result, for example, 0–36. * for persons with dementia, *p* < 0.003 was regarded as significant and for informal caregivers *p* < 0.008 was regarded as significant; significant *p*-values are marked in bold.

**Table 4:** Multivariate regression analysis of associated factors of people with dementia and their caregivers.

Urban and Rural Area	Adjusted R^2^	OR	95% CI	*p* ≤ 0.05
Living at home				
Person with dementia				
-		-	-	-
Informal caregiver	0.066			
Caregiver burden		1.033	1.001–1.066	**0.043**
Living in a nursing home				
Person with dementia				
-		-	-	-
Informal caregiver	0.193			
Age		1.072	1.019–1.127	**0.007**

CI = confidence interval; OR = odds ratio. *p* ≤ 0.05 was regarded as significant, significant values are marked in bold. * Nagelkerke R Square.

## Supplementary Materials

The following are available online at https://www.mdpi.com/2227-9032/7/2/80/s1, Table S1: Descriptive data of the inhabitants in the participating municipalities (as at 31 December 2014) and the study’s attrition rate.
