# Supplementary material for: Persons with Dementia Living at Home or in Nursing Homes in Nine Swedish Urban or Rural Municipalities
Source: Healthcare (Basel). 2019 Jun 25;7(2):80. doi: 10.3390/healthcare7020080 (PMC6627377; doi:10.3390/healthcare7020080)
Supplement: Supplementary file 1 [file healthcare-07-00080-s001.pdf]

Table S1. Descriptive data of the inhabitants in the participating municipalities (as at 31 December 2014), and the study´s attrition rate.

**Municipality**	**Inhabitants**	**Inhabitants ≥65 years (%)**	**Inhabitants ≥85 years (%)**	**Living in Rural Areas (%)**	**Attrition Rate HC/NH (%)**
1 *	28,737	5778 (20)	833 (3)	20	89/60
2	8256	1943 (24)	289 (4)	13 **	86/57
3 *	27,522	6294 (23)	921 (3)	26	91/55
4 *	9549	2475 (26)	340 (4)	22	87/55
5	12,198	3380 (28)	574 (5)	36	86/57
6	9222	2260 (25)	382 (4)	23	86/58
7	86,970	15,934 (18)	2,235 (3)	12	83/60
8 *	15,908	3379 (21)	560 (4)	25	88/57
9	19,505	4159 (21)	653 (3)	28	100/60

HC = home care; NH = nursing homes. * Rural area. ** Most people were living in small villages not located in rural areas.
